# Use of chitosan and β-tricalcium phosphate, alone and in combination, for bone healing in rabbits

**DOI:** 10.1007/s10856-013-5091-2

**Published:** 2013-11-17

**Authors:** A. S. Azevedo, M. J. C. Sá, M. V. L. Fook, P. I. Nóbrega Neto, O. B. Sousa, S. S. Azevedo, M. W. Teixeira, F. S. Costa, A. L. Araújo

**Affiliations:** 1 Unidade acadêmica de Medicina Veterinária (UAMV), Centro de Saúde e Tecnologia Rural (CSTR), Universidade Federal de Campina Grande (UFCG), Av. Universitária s/n, Bairro Santa Cecília, Patos, PB 58700-970 Brazil; 2 Unidade Acadêmica de Engenharia de Materiais (UAEM), UFCG, Campina Grande, PB Brazil; 3 Departamento de Medicina Veterinária (DMV), Universidade Federal Rural do Pernambuco (UFRPE), Recife, PE Brazil

## Abstract

The aim of this research was to evaluate the process of bone regeneration in rabbits, using chitosan and beta-tricalcium phosphate (β-TCP) independently and in combination. A total of 12 New Zealand rabbits of both sexes, with average weight of 3.0 ± 0.57 kg were used. Animals were randomly divided into two experimental time points, with six animals euthanized 45 days after surgery and six euthanized 90 days after surgery. We performed two osteotomies in each tibia. The left tibia was used for the chitosan (QUI) and control groups, and the right tibia was used for the β-TCP alone and in combination with chitosan (QUI+TCP) groups. Tomographic evaluation showed no statistically significant difference among groups; however radiopacity was higher in the treated groups. Comparative descriptive histological evaluation found that treatment groups stimulated a more pronounced tissue repair reaction and accelerated bone repair. Morphometric analysis showed that treatment groups presented statistically higher bone formation compared with the control group.

## Introduction

The use of biomaterials in orthopedic surgery is becoming routine, allowing for greater option in cases requiring an adjuvant for the process of bone repair. Among the list of biomaterials, calcium phosphates are widely studied and used in dentistry and bone surgery [1, 2]. Beta-tricalcium phosphate (β-TCP), compared with hydroxyapatite, is more osteoconductive with excellent biodegradation properties, characterized by rapid absorption and replacement by new bone matrix [3, 4]. Several studies investigating biopolymers highlight the successful use of chitosan, due to its ability to stimulate bone induction, thus favoring faster repair [5]. This can be explained by the unique surface property of chitosan, which stimulates macrophage receptors, thus promote the release of growth factors, maximizing the osteogenic process [6–8]. Several combinations of biomaterials have been studied to accelerate and maximize the regeneration of bone tissue [9]. The combination of chitosan with β-TCP provides an alternative reparative process for bone tissue. This composite provides, not only a stimulus for bone induction from chitosan, but also osteoconductivity from the β-TCP mineral, thereby favoring increased formation of bone matrix by osteoblasts, as supported by previous studies [5, 10].

The objective of this study was to evaluate the bone regeneration process, stimulated by the use of chitosan and β-TCP, independently and combined, in rabbit tibia defects.

## Materials and methods

This experimental study was approved by the Ethics Committee in Research of the Veterinary Medicine Academic Unit, at the Federal University of Campina Grande (UAMV/UFCG) according to the approved protocol No. 10/2012.

### Animals

In this experiment 12 New Zealand adult rabbits (mean weight 3.0 ± 0.57 kg) of both sexes were used. The animals were randomly divided into two experimental time points, with six animals in each time group, according to the period of euthanasia (45 or 90 days after surgery). Animals were placed in individual cages, dewormed with albendazole (5 % Ibazole-IBASA) by giving 20 mg/kg orally and passed through an adjustment period of 7 days before the start of the experiment. Animals received balanced rations twice a day and were given drinking water ad libitum throughout the experiment.

### Synthesis and characterization of implants

The implants used in this study were provided by the Biomaterials Group, Department of Materials Engineering at the UFCG. For preparation of the sponge implants, chitosan (Sigma-Aldrich, Brazil, 448877, molecular weight 1.90 × 10^5^–3.10 × 10^5^ g/mol, deacetylation degree 75–85 %), glacial acetic acid (Vetec, Brazil) and genipin (Sigma-Aldrich, Brazil, 6902778, molecular weight 226.23 g/mol, purity ≥98 % HPLC) were used. For further processing of scaffolds, absolute ethanol 99.5 % GL (Nuclear, Brazil), hydrated alcohol 70 % INPM (TUPI, Brazil) and sodium hydroxide (Vetec, Brazil) were used.

For the synthesis and characterization of β-TCP, the method of precipitation by wetting, involving a neutralization reaction between phosphoric acid solution (H_3_PO_4_) and calcium hydroxide [Ca(OH)_2_] was used. The stoichiometric amounts of the solutions were determined according to the value of the atomic ratio between the calcium and phosphorus atoms from calcium phosphate. Powder Ca(OH)_2_ was added to deionized water and vigorously agitated and heated to 80 °C. To this solution, H_3_PO_4_ was slowly added under constant agitation. After thorough mixing of the two reactants, the temperature was raised to 100 °C and agitation was continued until viscosity was reached. The ceramic paste obtained was dried at 110 °C for 24 h and the product was deagglomerated, passed through a 200 mesh sieve to obtain the powder, heat-treated at 20 °C per minute and held at 1,100 °C for 2 h.

### Surgical procedure

After shaving, a pre-anesthesia medication of acepromazine (Acepran 1 %, VETNIL, Brazil), 1 mg/kg was given intravenously (IV), and then anesthesia medication comprising tiletamine associated with zolazepam (Zoletil 100, VIRBAC, Brazil) was given at a dose of 15 mg/kg IV. We also performed epidural anesthesia with 2 % lidocaine (anesthetic BRAVET, BRAVET, Brazil) at a dose of 0.22 mL/kg, associated with tramadol (Tramal-PFIZER, Brazil) at a dose of 1 mg/kg. After antisepsis of the operative area with a solution of chlorhexidine 0.5 % (0.5 % Riohex, RIOQUÍMICA, Brazil), a skin incision was made along the medial margin of the tibial crest and dilatation of the subcutaneous tissue and muscle was carried out. Longitudinal resection of the periosteum was performed and two holes were constructed, one in the proximal metaphysis and another at the distal metaphysis, using a surgical drill with a 3.0-mm diameter for implant placement. Treatments were as follows, in the left tibia; control group (C), osteotomy was performed but not completed for any implant in the proximal metaphysis; and chitosan group (QUI) with 85 % deacetylation on the distal metaphysic; and in the right tibia; the β-TCP group (TCP) in the proximal metaphysis was introduced at a Ca/P ratio of 1.5, and the β-TCP combined with chitosan group (QUI+TCP) on the distal metaphysis. All implants were autoclaved prior to use. Synthesis was performed in the tissues. This procedure was performed in both limbs.

### Postoperative

Postoperatively, animals received enrofloxacin (2.5 % Biofloxacin, BIOVET) at a dose of 10 mg/kg intramuscularly (IM), once a day for 5 days and meloxicam (0.2 % Maxicam, FINE GOLD) at a dose of 0.2 mg/kg IM on the first day and 0.1 mg/kg in two subsequent days. Cleaning of the wound was performed with saline and Kuraderm Silver (Silver Kuraderm, AVIPEC) during the first 10 days after surgery and stitches were removed thereafter.

### Computerized tomography examination

At the end of the observation period for each experimental time point, tibias were harvested and computerized tomography (CT) was performed, with a helical unit GE Hi-Speed FXI and protocol with 120 kVp and auto mA, at rotation speed of one per second. Images were acquired in transverse sections 1 mm thick with a filter for bony parts. After the CT scan and image processing, the amount of attenuation in Hounsfield units (HU) of the bone was calculated from the average of three regions of interest. Each region of interest had its area previously standardized for better uniformity of results. The software for tomographic analysis was the E-film.

### Histologic evaluation of the bone/implant interface

For microscopic evaluation, tibiae were harvested and the fragments of bone containing the implant were removed. The bone fragments were chemically fixed with 10 % buffered formalin for 10 days. Shortly after, the material was washed with water and demineralized with a mixture of equal parts of 5 % formic acid + 5 % hydrochloric acid for 14 days. Fragments were embedded in liquid paraffin and sliced at a thickness of 5 μm transverse blocks and mounted on slides. From each block four slides were obtained and subjected to hematoxylin–eosin staining for histological observation. The purpose for these observations was to evaluate the bone–implant interface degrees of endosteal and periosteal reaction, cell proliferation, differentiation and healing of the scar tissue on the bone lesion. In this evaluation, we performed descriptive qualitative assessment and comparison between times and groups.

### Morphometric analysis of the bone/implant interface

Osteogenesis, induced by treatment, was quantified by morphometric analysis of slides through Image Pro Plus^®^ Version 6.2. For these analyses, images that comprised bone/implant interface were captured and processed. Sequential images of each slide were analyzed to quantify, in μm^2^ and mm^2^, new bone at the implant interface. Average values were obtained for every group studied and statistical analysis was performed.

### Statistical evaluation

A comparison of bone regeneration, induced by treatments, was carried out for each experimental time point and between experimental groups. Initially we performed the Anderson–Darling normality test for verification of the data distribution. For normally distributed variables, the groups were compared by one-way analysis of variance (ANOVA), with multiple comparisons by Tukey’s test. For variables with non-normal distribution, comparison was performed by the nonparametric Kruskal–Wallis test with multiple comparisons by the Nemenyi test [11]. The significance level was 5 % and the analyses were performed with the statistical program MINITAB Version 14.0.

## Results

### CT evaluation

**Fig. 1:**
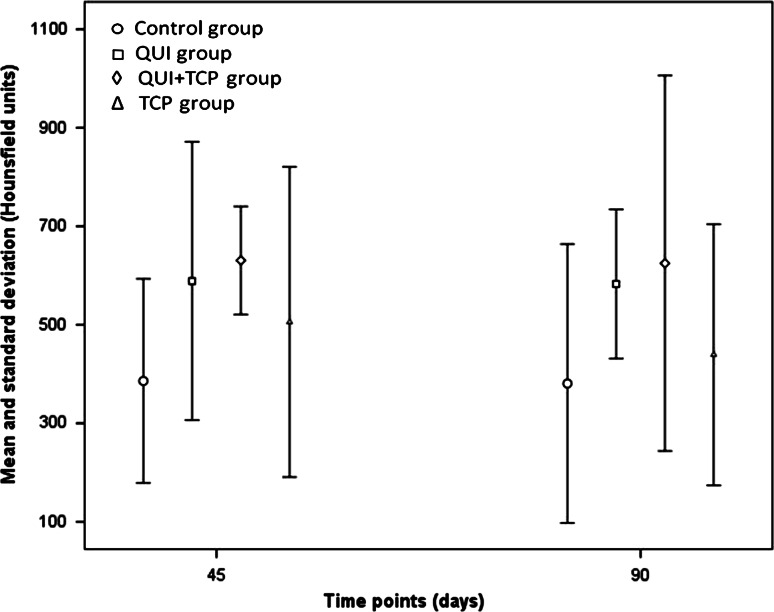
Mean and standard deviation of radiodensity in Hounsfield units (HU) at 45 and 90 days. Osteotomized area received the treatments; *C* control, *QUI* chitosan only, *QUI+TCP* chitosan in combination with tricalcium phosphate (TCP), *TCP* TCP only

CT evaluation found no statistical difference between groups or between times. However, as highlighted in Fig. 1, the mean HU values for the groups, TCP+QUI, QUI and TCP, were higher in the experimental time points studied compared with group C.

### Histological descriptive evaluation

**Fig. 2:**
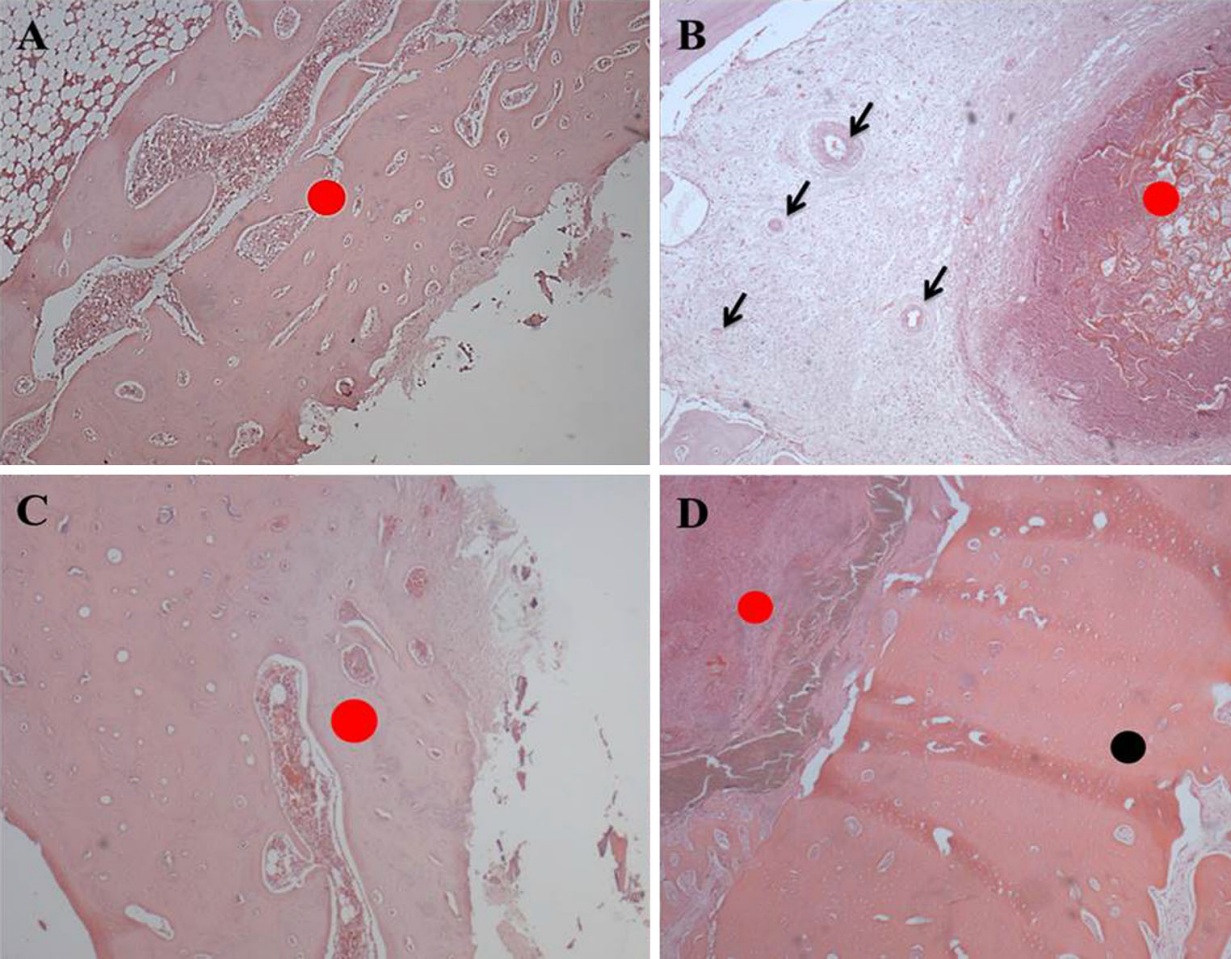
Descriptive histological evaluation at day 45. **a** (Control group); *red circle* indicates osteotomized site filled by newly formed immature bone. **b** (QUI group); *red circle* shows implantation of chitosan surrounded by intense cellular reaction, *dark arrows* highlight blood vessels. **c** (TCP group); *red circle* indicates osteotomized site filled by newly formed bone tissue. **d** (TCP+QUI group); *red circle* indicates the implant site with intense cellular reaction around the implant and *black circle* highlights osteotomized area filled by newly formed bone tissue. Obj. ×4 (Color figure online)

Following histological evaluation, it was observed that the bone/implant interface for groups QUI, TCP and QUI+TCP, showed higher cell reaction of granuloma type than group C at day 45. A greater presence of neovascularization and biomaterial–cell interaction in bone formation was also observed in the center of the granulomas in the same groups. This reaction was more intense in groups QUI+TCP and QUI (Fig. 2). Furthermore, a greater amount of new bone on the bone/implant interface was observed in the QUI and QUI+TCP groups compared with the other groups and the reactions of the TCP group were higher compared with group C.

**Fig. 3:**
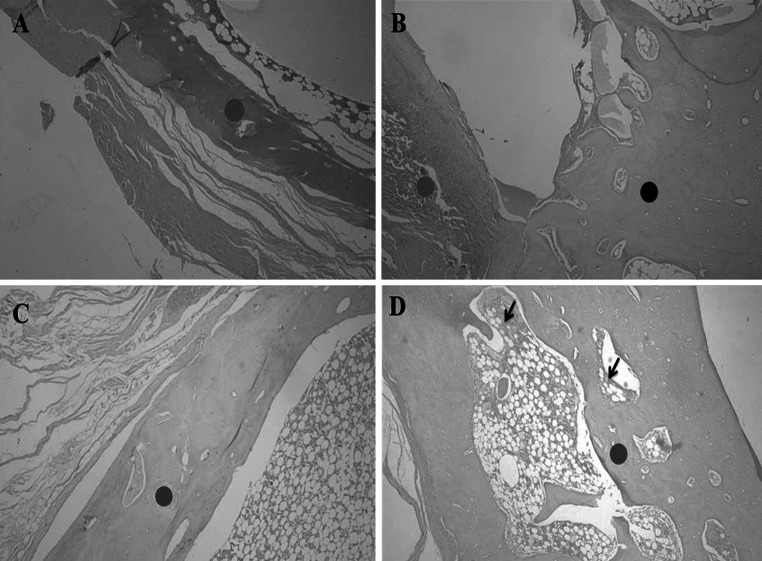
Descriptive histological evaluation at day 90. **a** (Control group); *red circle* indicates osteotomized site filled by newly formed immature bone with little cellular reaction. **b** (QUI group); *red circle* shows implantation of chitosan surrounded by intense cellular reaction and* dark circle* shows newly formed bone tissue. **c** (TCP group); *red circle* indicates osteotomized site filled by newly formed bone during the organization process. **d** (TCP+QUI group); *red circle* indicates the osteotomy site filled by newly formed bone, in the organization process, and *black arrows* show cellular reaction. Obj. ×4 (Color figure online)

Histological evaluation at day 90, revealed the bone/implant interface of QUI and QUI+TCP groups showed a higher cellular reaction with granuloma than the other groups. It also showed an intense neovascularization in those groups, which was less intense in group C. It was observed that the biomaterials were still present and surrounded by cells. All groups had advanced bone healing, but in groups QUI and QUI+TCP, this reaction was more pronounced than in the others (Fig. 3).

### Morphometric evaluation

**Fig. 4:**
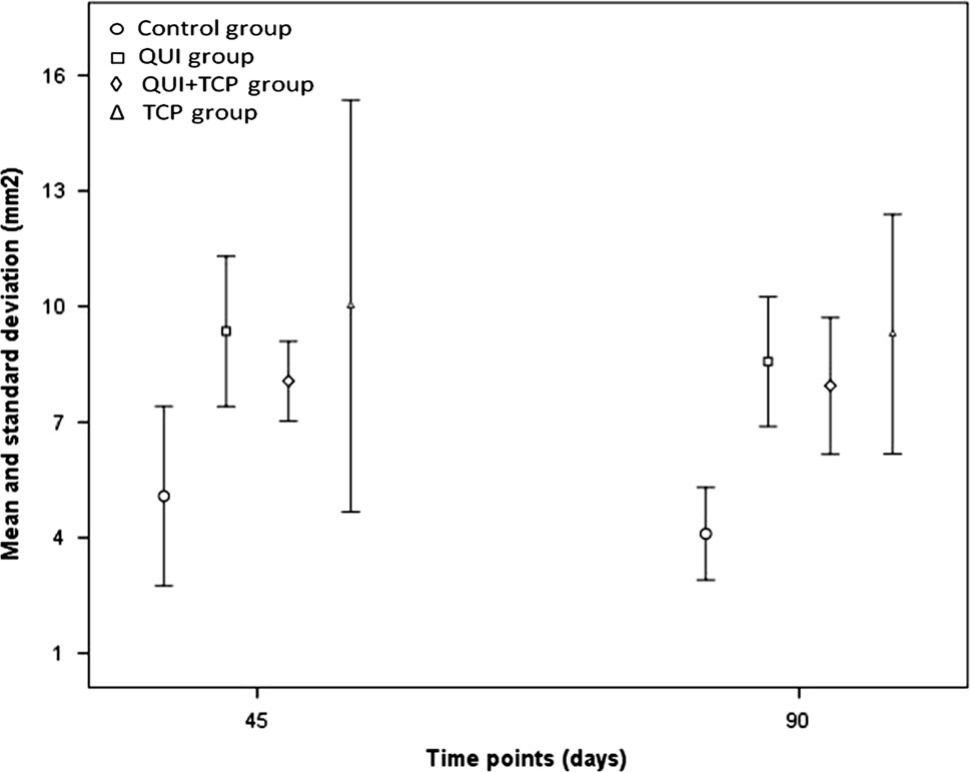
Mean and standard deviation of newly formed bone tissue (mm^2^) at the treatment interface. Treatments; *C* control, *QUI* chitosan only, *QUI+TCP* chitosan combined with tricalcium phosphate, *TCP* TCP only)

Morphometric evaluation of the slides revealed a statistical difference (*P* = 0.036) between groups C and QUI at day 45. At day 90, there were statistical differences between groups C and QUI (*P* = 0.001), C and QUI+TCP (*P* = 0.0005) and C and TCP (0.003) as shown in Fig. 4.

## Discussion

Tomographic evaluation of bone tissue is an applicable and reliable research method for measuring bone density [12]. In the present study, there was no statistical difference between groups; however groups QUI, QUI+TCP and TCP obtained mean radiopacity higher than group C. Even in the absence of a statistical difference, possibly owing to the limited number of animals used in this study, there was a trend of greater radiopacity in all groups when compared with group C. This finding can be explained by the characteristics of chitosan, which is an osteoinductive material that stimulates the release of growth factors, differentiation and cell aggregation in the wound, thereby promoting and accelerating the regeneration of bone tissue [13]. When combined with the characteristics of β-TCP, an osteoconductive material that acts as a substrate, providing minerals that favor mineralization of the extracellular matrix, thereby stimulating osteogenesis [14], it is not surprising the treatments in this study advance bone formation compared with the control.

Descriptive histological evaluation in both the 45 and 90 day groups for QUI, QUI+TCP and TCP, identified more intense and evident reactions compared with group C. It could also be noted that groups QUI and QUI+TCP showed greater reactions compared with the TCP group. In this study, the characteristic wider and larger reactions after treatment can be attributed to the characteristics of chitosan, which stimulates the release of growth factors and cell differentiation, increasing and maximizing osteogenesis [7–15]. Together with TCP, which promotes mineralization of bone matrix, faster tissue healing occurs [16, 17]. Previous studies using different calcium phosphates have highlighted this [9]. The combination of biopolymer with calcium phosphate in promising [5], in enabling the materials to synergistically promote cell differentiation and accelerate deposition of osteoid in the bone defect, therefore accelerating the process of osteogenesis [18]. This was observed in our study, whereby broad cellularity and bone formation was evident after treatment compared with the control.

Morphometric analysis, identified no statistically significant difference at day 45 between groups C and QUI and at day 90 between groups C and QUI, QUI+TCP and TCP. In the present study, materials containing chitosan and β-TCP stimulated bone formation to greater extent compared with the control group. This can be explained by the ability of chitosan to stimulate release, at the injury site, of interleukins 1, 6 and 8, other inflammatory factors and macrophages, all of which accelerate the process of tissue repair by stimulating osteogenesis [6], as observed in this study. In a recent study, chitosan was shown to stimulate bone formation tenfold higher compared with calcium phosphate [19]. When used alone or combined with chitosan, β-TCP provides calcium and phosphorous ions, which enable osteoblasts to synthesize osteoid, thus enhancing the bone repair process. Furthermore, calcium phosphate is rapidly absorbed and stimulates deposition of osteoid in the same proportion [9]. In the present experiment, we observed that chitosan alone or combined with β-TCP favors osteogenesis, with enhanced bone formation in bone lesions.

## Conclusion

We conclude that combined chitosan and β-TCP used in this study stimulates osteogenesis to a greater extent compared with the control group. The combination of these biomaterials is very promising in the future repair of bone tissue.
